# Supplementary material for: Characterization of the Chloroplast Genome of Argyranthemum frutescens and a Comparison with Other Species in Anthemideae
Source: Genes (Basel). 2022 Sep 25;13(10):1720. doi: 10.3390/genes13101720 (PMC9602088; doi:10.3390/genes13101720)
Supplement: Supplementary file 1 [file genes-13-01720-s001.zip › genes-1904567-supplementary/supplementary file/supplementary information.pdf]

Characterization of the chloroplast genome of Argyranthemum frutescens and a comparison with other species in Anthemideae

Yiran Zhao, Danyue Qu, Yueping Ma*

College of Life and Health Sciences, Northeastern University, Shenyang, 110004, China

* Correspondence: Yueping Ma, mypluna@sina.com

Table S1. Codon usage in chloroplast genome of Argyranthemum frutescens.

AA	codon	Freq	RSCU	AA	codon	Freq	RSCU
Phe	UUU	957	1.32	Ser	UCU	576	1.76
	UUC	495	0.68		UCC	315	0.96
Leu	UUA	872	1.89		UCA	395	1.21
	UUG	554	1.2		UCG	153	0.47
	CUU	616	1.33	Pro	CCU	435	1.58
	CUC	182	0.39		CCC	185	0.67
	CUA	357	0.77		CCA	320	1.16
	CUG	189	0.41		CCG	160	0.58
Ile	AUU	1076	1.47	Thr	ACU	527	1.63
	AUC	427	0.58		ACC	238	0.74
	AUA	696	0.95		ACA	404	1.25
Met	AUG	612	1		ACG	123	0.38
Val	GUU	491	1.45	Ala	GCU	607	1.72
	GUC	166	0.49		GCC	222	0.63
	GUA	520	1.53		GCA	414	1.17
	GUG	180	0.53		GCG	168	0.48
Tyr	UAU	800	1.64	TER	UAA	49	1.73
	UAC	176	0.36		UAG	21	0.74
Cys	UGU	196	1.37		UGA	15	0.53
	UGC	90	0.63	Trp	UGG	447	1
His	CAU	449	1.52	Arg	CGU	338	1.33
	CAC	143	0.48		CGC	100	0.39
Gln	CAA	705	1.51		CGA	339	1.33
	CAG	229	0.49		CGG	116	0.45
Asn	AAU	975	1.56	Ser	AGU	403	1.23
	AAC	276	0.44		AGC	120	0.37
Lys	AAA	1021	1.49	Arg	AGA	470	1.84
	AAG	347	0.51		AGG	167	0.65
Asp	GAU	835	1.59	Gly	GGU	580	1.32
	GAC	213	0.41		GGC	187	0.42
Glu	GAA	987	1.5		GGA	689	1.57
	GAG	331	0.5		GGG	305	0.69

Table S2. Putative RNA Editing Sites of Argyranthemum frutescens chloroplast genome.

CDS	Nt Pos	AA Pos	Align Col	Effect	Score
matK	284	95	108	TCT (S) => TTT (F)	0.86
	637	213	229	CAT (H) => TAT (Y)	1
	1240	414	430	CAT (H) => TAT (Y)	1
rpoB	983	328	345	GCT (A) => GTT (V)	1
rpoC1	511	171	171	CCC (P) => TCC (S)	1
	802	268	284	CGT (R) => TGT (C)	1
	1592	531	548	GCA (A) => GTA (V)	0.86
	2045	682	710	CCA (P) => CTA (L)	1
rpoC2	1960	654	835	CTT (L) => TTT (F)	0.83
	2725	909	1106	CCC (P) => TCC (S)	1
	3716	1239	1454	TCG (S) => TTG (L)	0.86
rps2	248	83	83	TCA (S) => TTA (L)	1
atpI	629	210	213	TCG (S) => TTG (L)	1
atpA	773	258	258	TCA (S) => TTA (L)	1
rps14	80	27	27	TCA (S) => TTA (L)	1
	149	50	53	CCA (P) => CTA (L)	1
atpB	1469	490	496	TCG (S) => TTG (L)	1
accD	125	42	42	ACG (T) => ATG (M)	1
	301	101	132	CCT (P) => TCT (S)	1
	1162	388	450	CCA (P) => TCA (S)	1
	1370	457	519	CCT (P) => CTT (L)	1
psbL	2	1	1	ACG (T) => ATG (M)	1
psbF	77	26	26	TCT (S) => TTT (F)	1
petG	14	5	5	TCT (S) => TTT (F)	0.86
petB	418	140	140	CGG (R) => TGG (W)	1
	611	204	204	CCA (P) => CTA (L)	1
petD	305	102	102	GCT (A) => GTT (V)	0.86
rpoA	824	275	279	TCA (S) => TTA (L)	1
ndhB	149	50	50	TCA (S) => TTA (L)	1
	259	87	87	CTT (L) => TTT (F)	1
	467	156	156	CCA (P) => CTA (L)	1
	586	196	196	CAT (H) => TAT (Y)	1
	611	204	204	TCA (S) => TTA (L)	0.8
	737	246	246	CCA (P) => CTA (L)	1
	746	249	249	TCT (S) => TTT (F)	1
	830	277	277	TCA (S) => TTA (L)	1
	836	279	279	TCA (S) => TTA (L)	1
	1481	494	494	CCA (P) => CTA (L)	1

ndhA	107	36	36	CCT (P) => CTT (L)	1
	566	189	189	TCA (S) => TTA (L)	1
	1073	358	358	TCC (S) => TTC (F)	1
ndhG	166	56	56	CAT (H) => TAT (Y)	0.8
	314	105	105	ACA (T) => ATA (I)	0.8
ndhD	2	1	1	ACG (T) => ATG (M)	1
	383	128	128	TCG (S) => TTG (L)	1
	599	200	200	TCA (S) => TTA (L)	1
	878	293	293	TCA (S) => TTA (L)	1
	887	296	296	CCC (P) => CTC (L)	1
	1310	437	437	TCA (S) => TTA (L)	0.8
ccsA	110	37	39	CCA (P) => CTA (L)	0.86
	370	124	127	CCC (P) => TCC (S)	0.86
ndhF	290	97	97	TCA (S) => TTA (L)	1
ndhB	149	50	50	TCA (S) => TTA (L)	1
	259	87	87	CTT (L) => TTT (F)	1
	467	156	156	CCA (P) => CTA (L)	1
	586	196	196	CAT (H) => TAT (Y)	1
	611	204	204	TCA (S) => TTA (L)	0.8
	737	246	246	CCA (P) => CTA (L)	1
	746	249	249	TCT (S) => TTT (F)	1
	830	277	277	TCA (S) => TTA (L)	1
	836	279	279	TCA (S) => TTA (L)	1
	1481	494	494	CCA (P) => CTA (L)	1

Note: Nt Pos shows the location of the nucleotide predicted to be edited in the input DNA sequence. AA Pos means the location of the amino acid predicted to be edited in the translation of the input DNA sequence. Align Col indicates the column in the AA alignment where the edit site occurs. Effect shows the codon and the encoded amino acid before and after editing. The prediction score is a value ranging from 0 to 1 and a higher value indicates more confidence.

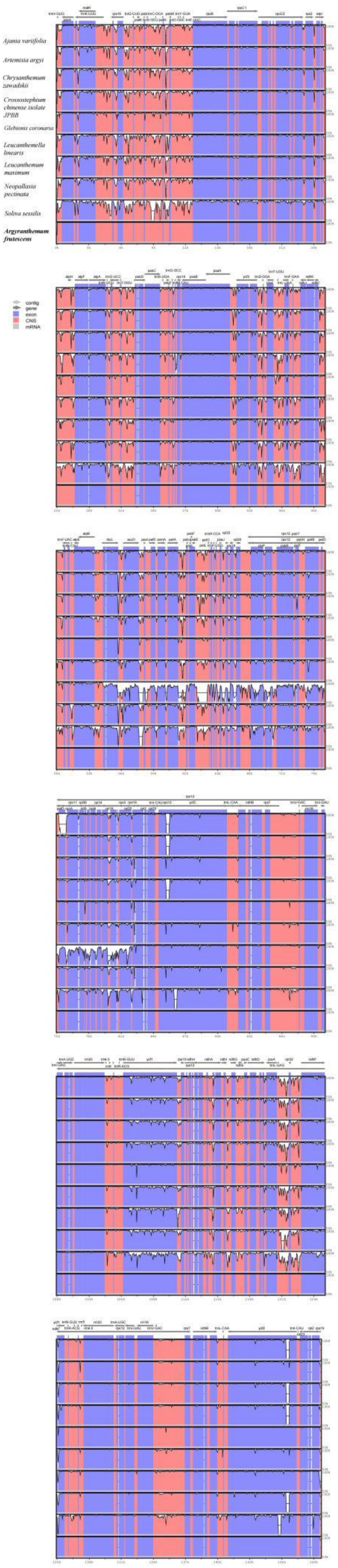

Figure S1. Sequence identity plots of the 10 Anthemideae chloroplast genomes generated by mVISTA. The vertical and horizontal axes in the figure represent the consistency degree of the sequences from 50% to 100% and the sequence length, respectively.
