# Supplementary material for: Characterization of the Chloroplast Genome of Argyranthemum frutescens and a Comparison with Other Species in Anthemideae
Source: Genes (Basel). 2022 Sep 25;13(10):1720. doi: 10.3390/genes13101720 (PMC9602088; doi:10.3390/genes13101720)

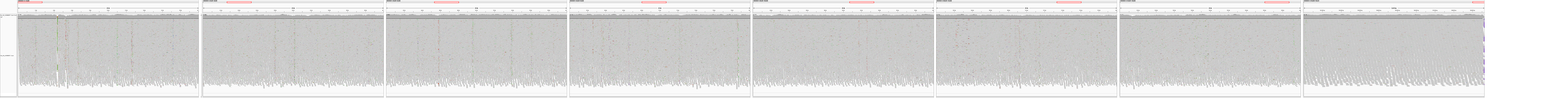

Supplement: Supplementary file 1 [file The assembly results by mapping short reads to the assembled sequence by Screen capture of the IGV.pdf › genes-1904567-supplementary/supplementary file/The assembly results by mapping short reads to the assembled sequence by Screen capture of the IGV.pdf]
